# Diuretic-Resistant Ascites Following Laparoscopic Surgery in a Patient With Chronic Myeloid Leukemia on Imatinib Therapy

**DOI:** 10.7759/cureus.13127

**Published:** 2021-02-04

**Authors:** Efthymia Pappa, Marina Gkeka, Ifigeneia Kiki, Pagona Gourna, Constantinos Christopoulos

**Affiliations:** 1 Internal Medicine, Sismanoglio-A. Fleming General Hospital, Athens, GRC; 2 Cardiology, Konstantopouleio-Patision General Hospital, Athens, GRC

**Keywords:** chronic myeloid leukemia, diuretic-resistant ascites, laparoscopic surgery, imatinib therapy

## Abstract

Imatinib mesylate is a tyrosine kinase inhibitor with high efficacy in the treatment of chronic myeloid leukemia (CML). Although fluid retention is a common adverse effect of imatinib, it rarely necessitates discontinuation of therapy. Isolated ascites has not been reported as a complication of imatinib therapy in patients with CML. Here, we report the case of a 72-year-old male with CML on imatinib (600 mg daily), who developed ascites two weeks after a laparoscopic hernia repair with intraperitoneal placement of a nylon mesh. The ascites was resistant to diuretic therapy and required repeated large-volume paracentesis. Discontinuation of imatinib resulted in arrest of ascites production, but reintroduction of the drug at the same dose two weeks later was rapidly followed by recurrence of ascites requiring further therapeutic paracenteses. It was postulated that peritoneal inflammation had resulted in increased capillary permeability, which was further augmented by imatinib via inhibition of platelet-derived growth factor receptor (PDGFR), a tyrosine kinase known to play a significant physiological role in the regulation of interstitial fluid pressure and capillary permeability. The possibility of developing ascites after abdominal surgery should be considered in patients receiving imatinib or related PDGFR inhibitors. In such cases, perioperative interruption of tyrosine kinase therapy might be indicated.

## Introduction

The advent of imatinib, a tyrosine kinase inhibitor (TKI) that blocks the action of the BCR/ABL fusion protein, has dramatically changed the prognosis for chronic myeloid leukemia (CML) patients, who can now expect long-term survival with a good quality of life. Fluid retention is among the most common, clinically relevant adverse effects of imatinib therapy. It usually manifests as superficial edema not requiring drug cessation, although severe generalized edema with serosal effusions and anasarca may occur on rare occasions, especially in patients treated with high doses of the drug (>400 mg daily). In such cases, discontinuation of therapy may be necessary [1-3].

Isolated ascites has not been reported in the setting of imatinib therapy for CML. Here, we present the case of a CML patient on long-term imatinib therapy, who developed diuretic-resistant, imatinib-dependent ascites following the placement of an intraperitoneal nylon mesh for repair of an umbilical hernia. The case offers insights into the pathogenesis and management of imatinib-induced ascites.

## Case presentation

A 72-year-old male was referred to our department with an eight-week history of furosemide-resistant ascites which had appeared two weeks after a laparoscopic umbilical hernia repair. His medical history was notable for CML diagnosed nine years ago. His disease was in major molecular remission (BCR-ABL/ABL ratio <0.1%) on imatinib mesylate 600 mg daily. The drug was generally well tolerated, the only adverse effects being occasional muscle cramps and skin discoloration. The laparoscopic hernia repair operation had been uneventful. It involved adhesiolysis of multiple adhesions, reduction of omentum protruding through the hernia, and intraperitoneal placement of a 10 × 15 cm polypropylene mesh covered with omega-3 fatty acids (C-Qur™).

**Figure 1:**
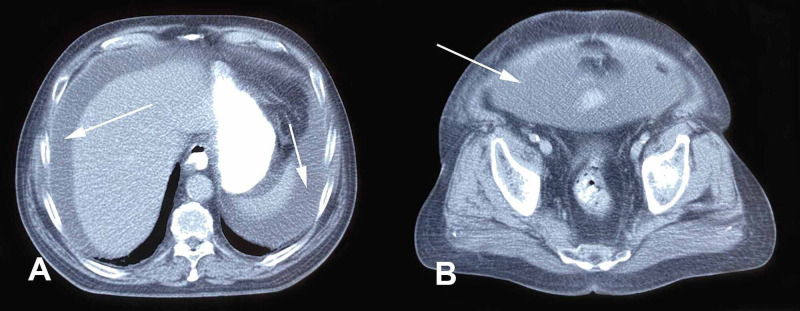
CT of the upper (A) and lower (B) abdomen showing the presence of free fluid (arrows). CT, computerized tomography

On physical examination, the patient was apyrexial and hemodynamically stable. There was abdominal distention with shifting dullness to percussion, but he had no peripheral edema or signs of pleural effusion. Abdominal ultrasound examination confirmed the presence of ascites. Diagnostic paracentesis revealed an exudate with serum-ascites albumin gradient of 0.7 and an ascites/serum lactate dehydrogenase ratio of 0.74. The fluid contained 730 cells/mL (38% lymphocytes, 23% neutrophils, 22% monocytes, 17% atypical). Gram stain and culture of ascitic fluid were negative, while adenosine deaminase levels were not increased. A full blood count showed a hemoglobin of 10.4 g/dL with normal white cell and platelet counts. Serum C-reactive protein levels were 33.2 mg/L (normal <3.5). A serum biochemistry profile showed only mild elevation of creatinine (1.5 mg/dL), which returned to normal following cessation of diuretic therapy. Urinalysis, plain chest radiogram, and electrocardiogram showed no abnormalities. Echocardiogram showed normal ejection fraction. Computerized tomography of the abdomen confirmed the presence of free fluid without additional abnormalities (Figures 1A, 1B).

Because of symptomatic tense ascites, the patient was managed with repeated large-volume therapeutic paracentesis. As the ascitic fluid was reaccumulating rapidly, imatinib was discontinued for two weeks, resulting in spectacular arrest of ascites production. Subsequent reintroduction of imatinib at the same dose (600 mg daily) was rapidly followed by recurrence of exudative ascites, requiring further therapeutic paracenteses. Again, pausing imatinib administration was effective in preventing ascites production. After two weeks, the drug was reintroduced at a low dose (200 mg daily) escalating to a maintenance dose of 400 mg daily over a period of a month without reappearance of ascites. Two years later, the patient remains free of ascites, while his CML continues to be in major molecular remission.

## Discussion

Severe fluid retention including pleural and pericardial effusions, ascites, and anasarca has been reported to occur in less than 1% of chronic-phase CML patients receiving imatinib [1,3]. It can occur shortly after the onset of treatment or later in the course of imatinib therapy and is associated with older age, female sex, higher drug dose, and comorbidities, especially renal and cardiac disease [1,2]. In our patient, application of the Naranjo adverse drug reaction probability scale gave a score of 10, signifying a definite causal relation of imatinib therapy to ascites development. However, given that the patient had been taking the same dose of imatinib for a long time without clinical evidence of fluid retention, the occurrence of ascites two weeks after the hernia repair suggests that the surgical procedure might have contributed to its pathogenesis. Conversely, the rapid disappearance of ascites upon discontinuation of the drug and the immediate relapse when treatment was reinstituted imply that surgery alone could not have caused this complication.

The mechanism of imatinib-induced edema is unclear. As with other adverse effects of the drug, off-target inhibition of tyrosine kinases (TKs) in normal tissues has been incriminated. Platelet-derived growth factor receptor (PDGFR), a TK inhibited by imatinib at therapeutic doses, is known to play a role in the regulation of interstitial fluid pressure (P_if_) and capillary permeability [4]. Inhibition of PDGFR abolishes the interaction between platelet-derived growth factor (PDGF) and cell adhesion molecules (integrins) in the extrcellular matrix, resulting in lowering of P_if_ concomitant with edema formation [5]. In an in vitro study employing the EA.hy 926 line simulating endothelial cells, Vrekousis et al. showed that cell-to-cell cohesiveness was reduced, and intercellular permeability was increased by 2.76 folds in the presence of imatinib [6]. Hinchcliff et al. reported the development of fulminant capillary leak syndrome in a patient with scleroderma treated with imatinib [7]. The above arguments, notwithstanding the fact that only a small percentage of imatinib-treated patients develop severe edema, suggest that exposure to therapeutic concentrations of the drug may lower the threshold for edema formation but is not per se sufficient for the development of clinically significant fluid retention in interstitial tissues or body cavities.

Isolated imatinib-induced ascites has not been reported previously in CML patients. However, Posadas et al. have described the development of imatinib-induced, non-malignant ascites in a patient with ovarian carcinoma treated with a high dose of the drug (800 mg/day) [8]. They argued that, in such cases, ascites might be caused or augmented by imatinib and should be differentiated from malignant ascites caused by the underlying intraabdominal neoplasia. The implantation of intraperitoneal meshes for hernia repair is not associated with postoperative ascites. A mild, self-limiting foreign-body reaction is known to occur, and the use of fatty acid coating of the polypropylene mesh aims at reducing the inflammation and adhesion formation. Nonetheless, more severe reactions with exudate formation around the mesh have been occasionally described [9]. Peritoneal inflammation is associated with local vasodilation and increased microvascular permeability leading to edema and exudative ascites. Upregulation of PDGFR has been reported in areas of inflammation and is thought to be part of an edema-counteracting mechanism involving the interaction of PDGF with integrins and extracellular matrix [10,11]. Therefore, it can be postulated that, in the case of our patient, inhibition of PDGFR activation by imatinib resulted in amplified exudate production and formation of ascites.

## Conclusions

In conclusion, the possibility of postoperative development of ascites should be considered in patients who are receiving imatinib or related PDGFR inhibitors and are going to have abdominal surgery likely to cause a peritoneal inflammatory reaction. In such cases, the need for perioperative interruption of TKI therapy should be carefully examined on an individual patient basis. Ascites developing postoperatively in a patient receiving imatinib or another PDGFR inhibitor may respond to temporary withdrawal of the drug, after exclusion of other causes. Gradual reintroduction of the TKI, starting with a lower dose, may prevent ascites recurrence.
